# Return to Work: Managing Employee Population Health During the COVID-19 Pandemic

**DOI:** 10.1089/pop.2020.0261

**Published:** 2021-02-05

**Authors:** Maren S. Fragala, Zachary N. Goldberg, Steven E. Goldberg

**Affiliations:** Quest Diagnostics, Secaucus, New Jersey, USA.

**Keywords:** employee population health, COVID-19 pandemic, SARS-CoV-2, return to work practices, health benefits and costs

## Abstract

Coronavirus disease-2019 (COVID-19), caused by the severe acute respiratory syndrome coronavirus-2 (SARS-CoV-2), has abruptly transformed the outlook of employer health benefits plans for 2020 and 2021. Containing the spread of the virus and facilitating care of those infected have quickly emerged as immediate priorities. Employers have adjusted health benefits coverage to make COVID-19 testing and treatment accessible and remove barriers to care in order to facilitate the containment of the disease. Employers also are introducing strategies focused on testing, surveillance, workplace modifications, and hygiene to keep workforces healthy and workplaces safe. This paper is intended to provide evidence-based perspectives for self-insured employers for managing population health during the COVID-19 pandemic. Such considerations include (1) return to work practices focused on mitigating the spread of COVID-19 through safety practices, testing and surveillance; and (2) anticipating the impact of COVID-19 on health benefits and costs (including adaptations in delivery of care, social and behavioral health needs, and managing interrupted care for chronic conditions).

## Introduction

Coronavirus disease 2019 (COVID-19), caused by the severe acute respiratory syndrome coronavirus-2 (SARS-CoV-2), has created a pandemic and interrupted the world economy. The combination of large numbers of infections (more than 8.6 million people infected nationwide as of October 26, 2020^1^) and potential for severe illness and death has stimulated uncertainty for employee health programs. The uncertainty of the pandemic leaves employers to face several unknowns as businesses resume operations, employees return to work, and employers are managing immediate and near-term workforce health and business needs. In addition, with travel restrictions and remote work, employers are forced to adapt to continue business operations. The purpose of this article is to provide insights and considerations for managing population health as employees return to work, including: (1) return-to-work practices focused on mitigating the spread of COVID-19 through safety practices, testing and surveillance, and (2) anticipating the impact of COVID-19 on health benefits and costs (including adaptations in delivery of care, social and behavioral health needs, and managing interrupted care for chronic conditions).

## Background and Epidemiology

In the United States, early reports suggested that SARS-CoV-2 could infect 20%-60% of the population before the pandemic finishes its course.^2^ Each infected individual is believed to spread the infection to 2.2–3.58 others on average.^3^ As of October 26, 2020, 8.6 million cases and 224,000 COVID-19-related deaths have been reported in the United States according to the Johns Hopkins Coronavirus Resource Center^1^ and the Centers for Disease Control and Prevention (CDC).^4^ Infection fatality rates range from 0.5% to 3.6%, depending on whether asymptomatic cases are included.^5^

Most people (about 80%) recover from the disease, experiencing only mild symptoms and requiring no hospitalization.^6^ The CDC^7^ has issued guidance for managing COVID 19 at home, including isolation and symptom monitoring. However, older adults (ages ≥60 years) and those with underlying health conditions (eg, asthma, diabetes, heart disease) (>105 million Americans) have a higher risk of developing serious illness if they are infected with coronavirus. Severe complications include pneumonia in both lungs, organ failure, and death. More serious illness may require more extensive care such as hospitalization and respiratory therapy.

Vulnerability to severe COVID-19 symptoms may cause fear^8^ in individuals who are more vulnerable themselves or who care for those who are vulnerable when returning to work. Underlying disease,^9–11^ obesity,^12^ viral load,^13^ medications, overreaction of the immune response,^14^ and lung health^15,16^ may all relate to severity of COVID-19 symptoms. Yet, previously healthy younger adults (younger than age 50 years) also may experience severe COVID-19 (representing ∼5% of severe cases), including severe pneumonia, encephalitis, cardiovascular disease, and pediatric inflammatory multisystem syndrome.^17^

## Workplace Measures to Contain the Spread of COVID-19

The virus is believed to be spread by person-to-person contact via aerosolized respiratory droplets (released through talking, breathing, coughing, or sneezing) and touch, even from those not exhibiting symptoms.^18^ Airborne transmission in confined spaces (eg, airplanes, passenger cars, health care centers) also may be a means of transmission.^19,20^ Individuals with more severe illness may be more infectious because they tend to have higher viral loads and longer virus-shedding periods relative to those with milder illness.^13^ However, a higher viral load (and more severe case of COVID-19) is not always accompanied by symptoms, as high viral loads may be detected soon after illness onset, including in patients with minimal or no symptoms.^21^ High infection, morbidity, and mortality rates leave employers to worry about the spread of the disease among the workforce.

Employers are obliged to follow Occupational Safety and Health Administration (OSHA) standards to prevent occupational exposure to SARS-CoV-2, including personal protective equipment (PPE) standards and furnishing each worker with “employment and a place of employment which are free from recognized hazards that are causing or are likely to cause death or serious physical harm.”^22^ With low virus spread and high system preparation and capacity, businesses may prepare to resume or continue operations.

**FIG. 1.**
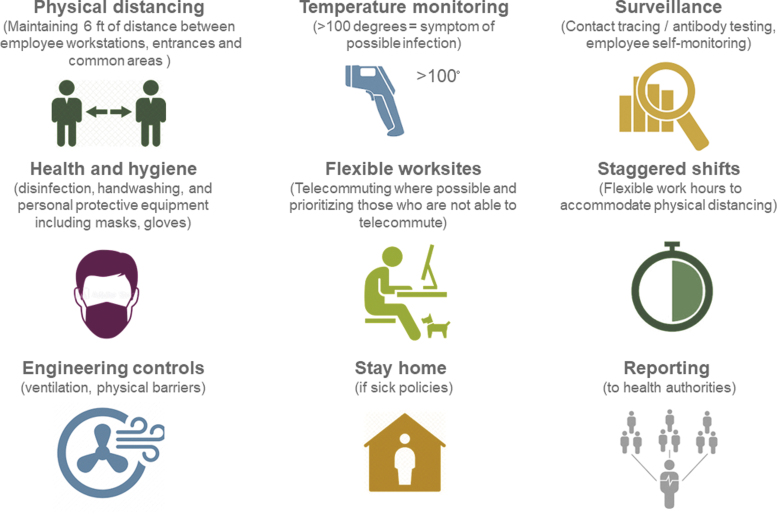
Workplace measures to contain the spread of COVID-19.^23,24^

As employers plan to resume business operations, the CDC,^23^ OSHA,^24^ and others have issued some guidance to attenuate the risk for further disease transmission. Key strategies for containing serious human outbreaks such as COVID-19 include (1) pharmaceutical countermeasures (eg, vaccines, antiviral medications), and (2) public health interventions (eg, infection control, social separation, quarantine).^25^ In the absence of sufficient medical countermeasures, public health measures have been the key strategy to contain the COVID-19 disease (Figure 1). Such measures include physical social distancing, symptom monitoring (temperature monitoring), hygienic measures (masks, disinfection procedures), disease surveillance and reporting, travel restrictions, quarantine, and case isolation.^26^ In addition, building engineering controls and workplace policies (flexible worksites, staggered shifts, sick policies) may aid in containing the spread. Reducing the rate of spread of the disease by public health interventions is necessary until medical countermeasures are developed to alleviate the strain on the health care system.^26^

### Source control via face mask use

Face coverings or masks could significantly contribute to controlling the pandemic, especially in conjunction with other nonpharmaceutical interventions that help to mitigate or suppress community transmission.^27^ Mask use suppresses the transmission of SARS-CoV-2 by preventing spread via respiratory droplets from infected to healthy individuals in community and health care settings.^28,29^ A systematic review and meta-analysis of 172 observational studies across 16 countries found that face mask use could result in a large reduction in risk of infection (risk difference of -14.3%).^30^ A meta-analysis of 21 studies found that mask use provided a significant protective effect against the spread of viral respiratory infections. These include a reduction in the risk of infection from influenza (45%), SARS (74%), and COVID-19 (96%).^31^ Thus, use of face coverings or masks is generally recommended at all times in places of employment when around other people.^24^

### Symptom screening

Symptom screening offers a tool to detect cases and contain the spread of the virus in a population.^32^ COVID-19 may be asymptomatic but also can cause mild to severe respiratory illness. Symptoms usually develop 3 to 7 days after exposure, but the incubation period may range up to 14 days.^33^ The most common symptoms include fever (over 100.4°F), fatigue, dry cough, and shortness of breath.^33,34^ Additional nonspecific or atypical symptoms include sore throat, diarrhea, myalgia (muscle aches, body aches) and fatigue.^35^ In addition to screening questionnaires for symptoms and known exposures, facilities may implement temperature screenings to screen staff,^36^ as elevated body temperature has been considered a main clinical finding of the viral infection.^37^ Although a feasible, noninvasive, and noncontact method to evaluate a symptom of the disease, it is best used in combination with evaluation of other symptoms to detect illness.^38^ Yet, symptom and temperature screening will only detect those experiencing such symptoms, and may miss approximately half of people infected because they have not yet developed symptoms and are unaware they were exposed.^32^ Nevertheless, isolation following onset of symptoms of COVID-19 may reduce SARS-CoV-2 transmission in the community up to 47%.^39^ Thus, daily symptom tracking (at home or in person) of employees before they enter the worksite and separation of those who exhibit signs or symptoms consistent with COVID-19 may be an important component of a workplace strategy to mitigate viral transmission.^40^

### Diagnostic testing

Testing for SARS–CoV-2 is necessary to identify individuals who are infected, both for their management and for mitigation strategies for the spread of the disease. The Equal Employment Opportunity Commission (EEOC) has advised that, in light of the COVID-19 pandemic, employers may choose to administer COVID-19 testing to employees before entering the workplace.^41^ As test results are considered private health information, test results and related employee certifications should be kept confidential.

Diagnostic testing for current infection requires identifying SARS-CoV-2 virus by either nucleic acid amplification or antigen directly from a patient's respiratory specimen (most commonly collected with an upper respiratory swab). Testing by nasal sampling of the upper or lower respiratory tract and direct detection of SARS–CoV-2 viral ribonucleic acid (RNA) through nucleic acid amplification by reverse transcription polymerase chain reaction (RT-PCR)^42^ is most commonly used to confirm diagnosis of COVID-19. Viral RNA-based tests are considered the best tests available to detect acute illness.^42,43^

Viral (PCR) testing can play an important role in prevention of SARS-CoV-2 transmission.^39^ Diagnostic testing may play an important role in confirming infection in those with symptoms, evaluating close contacts, and on a population level to contain the spread. Employees exhibiting COVID-19 symptoms are recommended to receive molecular testing and not return to work while awaiting test results in order to reduce the potential for transmission in the workplace.^40^ Contacts of employees with COVID-19 are also recommended prompt molecular testing, regardless of symptoms, using a risk-based approach based on likelihood of exposure.^40^ Testing should focus on those who work in the same area, on the same shift, in close proximity to one another, or in occupations with vulnerable populations (such as health care workers and other essential workers).^40^

### Population-based testing

Population-based testing of employees without symptoms or suspected exposure also may aid in early identification and transmission reduction, especially in locations with moderate to substantial community transmission.^40^ Asymptomatic or presymptomatic spread of COVID-19 occurs when infected people show no symptoms of COVID-19 and unknowingly infect others. As many as 35% of individuals who have COVID-19 are asymptomatic,^44,45^ accounting for approximately 40% to 45% of infections.^46^ In addition, about 44% of secondary cases of COVID-19 are infected during the index cases' presymptomatic stage, before symptoms develop.^18^ Focusing exclusively on symptomatic individuals for COVID-19 testing will inevitably miss asymptomatic patients capable of spreading the virus.^47^ Intermittent testing of asymptomatic individuals without known exposure to someone infected with COVID-19 may be required to stop transmission of the virus.^48,49^

Testing protocols may depend on access and availability of tests and observed positivity rates in the employee population and may prioritize initial testing of all employees before entering a workplace, testing of employees at regular intervals, and targeted testing of new employees and those returning from a prolonged absence.^40^ Population-based testing may be especially suited for workplaces where physical distancing is difficult (manufacturing, retail, education, service, maintenance) and critical infrastructure locations (health care, public safety, transportation, utilities, food and agriculture).^47,48^

CDC guidelines for early identification of asymptomatic employees in non-health care settings suggest regular testing of employees working in the same setting, and also suggest initial testing of all employees and testing of new entrants or those who have been absent from the work setting for a prolonged period.^40^ Frequency of testing may be established based on the availability of testing, latency between exposure and development of a positive SARS-CoV-2 viral test, rate of local community transmission, workplace positivity rates, and essential nature of job functions.^40^ Considering practical and financial constraints, population-level testing for COVID-19 infection surveillance may be prioritized for community “hot spots” where infection rates exceed, for example, a threshold of 10% of those tested.^50^

Weekly viral (PCR) screening of health care workers and other high-risk workers, regardless of symptoms, is estimated to reduce SARS-CoV-2 transmission by an additional 23% on top of reductions achieved by self-isolation following symptom identification.^39^ In addition, viral (PCR) testing of symptomatic individuals to identify SARS-CoV-2 infection also may reduce the number of individuals and contacts needing to self-isolate with confirmed negative test results.^39^

On a national level, population-based testing strategies have shown some success in containing the spread in countries such as South Korea.^51^ South Korea, for example, focused its strategy on rapid and widespread population-based testing, along with close contact tracing and isolation.^52,53^ As a result, South Korea has been able to contain the spread and toll of the disease.^54^ The effectiveness of such approaches may be attributable to the identification and isolation of asymptomatic carriers of the disease to prevent transmission.

Recommendations from the Harvard's Edmond J. Safra Center for Ethics report^55^ call for scaled up testing to 2%-6% of the US population per day in a targeted isolation approach in order to connect COVID-19-positive individuals to treatment and/or isolation to suppress the disease. As part of a population-based testing strategy, the University of Illinois at Urbana-Champaign, for example, implemented a strategy to test all of the students twice per week, meeting capacity with its own emergency use authorization-approved COVID-19 test.^56^ Efforts to accommodate COVID-19 test capacity are underway including, “pooled” testing.^57^ Since the start of the pandemic the daily quantity of tests performed nationally has increased from less than 300,000 in early May to more than 800,000 per day by July 2020.^1,58^ As of October 26, 2020, more than 140 million COVID-19 tests have been conducted on Americans, with 7% of tests positive (10.3 million).^59^

### Point-of-care diagnostic tests: RT-PCR

Although laboratory testing remains the primary testing mechanism for the nation, because of the ability to perform a high volume of tests at one time, point-of-care (POC) rapid tests for detection of SARS-CoV-2 RNA may provide additional testing options.^60,61^ POC tests typically run 1 sample at a time in 5–30 minutes in a facility-based platform and are thus not feasible for large populations. Yet, they may facilitate test access for populations who cannot readily access laboratory testing or need faster diagnoses to quickly address emerging outbreaks. For example, POC tests may be most appropriate for health care workers and individuals with high-priority symptoms (critically ill), while specimens from those without symptoms may be sent out for processing at an offsite laboratory using high-throughput platforms.^60^ Such tests also may play a role in maintaining essential workers (facilitating rapid return to work) or to conserve PPE usage.^60^ POC tests for SARS-CoV-2 (based on molecular or nucleic acid amplification) with emergency use authorization from the US Food and Drug Administration (FDA) include the Abbott ID NOW (Abbott Laboratories, Chicago, IL)^62^ and Cepheid GeneXpert Xpress (Cepheid, Sunnyvale, CA)^63^ (both are nucleic acid amplification tests).

Despite their convenience, POC tests may provide false-negative results because of the small viral particle size and the analytical process,^64,65^ consequently leading to more serious illness burden. The FDA has alerted the public to early data that suggest potential inaccurate results from using a POC test to diagnose COVID-19, where the tests were yielding false-negative results (May 2020).^64^ Thus, a subsequent laboratory-based PCR test may be performed for patients with negative results to aid in treatment or quarantine decisions.

### POC tests: antigen detection

Antigen POC tests detect fragments of viral proteins found in nasal cavity swabs within minutes. Although antigen tests are specific for the virus, they have lower sensitivity than molecular PCR tests and may miss identifying active virus.^66^ Thus, a negative antigen test result may not rule out infection and may need a follow-up PCR test prior to making treatment decisions or to prevent the possible spread of the virus.^60^ Antigen tests are best used with higher viral loads^66^ or when the virus is actively replicating—during acute or early infection.^67^ As of August 10, 2020, two antigen tests have received emergency use authorization from the FDA, including the Quidel Sofia 2 SARS Antigen FIA Veritor System for Rapid Detection of SARS-CoV-2 (Quidel Corporation, San Diego, CA).^68^

### Serological tests for antibodies

Blood-based (serological) tests can be used to identify whether a person has been previously exposed to SARS-CoV-2 and has developed an immune response. Individuals generate an immune response and neutralizing antibodies may be produced in response to SARS-CoV-2 infection. Antibodies to the virus are produced by plasma B cells initially. Immunoglobulin M (IgM) antibodies provide the first line of defense following viral infection. Subsequently, adaptive, high-affinity immunoglobulin G (IgG) antibodies are produced to provide long-term immunity and immune memory to specific viruses.^69^ IgG antibodies and neutralizing antibodies can block the virus from entering healthy cells and defend against viral reinfection. Eventually, longer lasting memory B cells also are stimulated to generate a more targeted and effective immune response to a subsequent exposure to the same virus. Some evidence suggests that neutralizing antibodies produced in response to prior SARS-CoV-2 infection can protect from reinfection,^70^ yet the duration of protective immunity remains unclear.

Studies of other coronaviruses may provide early insights into the immunity, management, and surveillance of SARS-CoV-2. For example, evidence from the first severe acute respiratory coronavirus (SARS-CoV) outbreak described in 2003 suggest that immune responses of specific antibodies were maintained for 2 years in more than 90% of recovered SARS-CoV patients.^71^ Presence of IgG antibodies has previously been shown to positively correlate with neutralizing antibodies to SARS-CoV.^72^ However, early evidence on SARS-CoV-2 immunity showed a rapid decline in IgG antibodies within 8 weeks in 93% (28/30) of individuals with asymptomatic COVID-19 and in 97% (30/31) of those with symptomatic illness.^73^ Conversion to seronegativity (seroreversion) for IgG was more common in patients with asymptomatic illness (12/30; 40%) than in patients with symptoms (4/31; 13%).^73^ In addition, some level of preexisting immunity may exist in the general population, given that 20%-50% of individuals who have not been exposed to SARS-CoV-2 appear to have T-cell reactivity against SARS-CoV-2.^74^

Presence of IgG antibodies may be indicative of some level of immune protection. Absence of immunoglobulin antibodies (immunoglobulin A, IgM, and IgG) suggests a person has not been exposed to SARS-CoV-2 or has been very recently and not yet generated an antibody response. Although interpretation of antibody tests requires caution, they may help in understanding acquired immunity to COVID-19, tracking exposures, and informing population-level exposures. Antibody testing provides important population-based data on pathogen exposure that can supplement detection of the transient active virus through RNA testing. Antibody testing can inform potential protective immunity in the population and guide vaccination strategies for the safe opening of communities and workplaces.^75^

### Specimen home collection for diagnostic testing

Self-collection of specimens at home provides a solution that allows people who suspect they have COVID-19 to get tested without exposing health care workers and others to the disease, and helps conserve PPE.^76^ Prior studies evaluating nasal swab self-collection for influenza testing has shown comparable results to professionally collected specimens.^77,78^ In one study, a majority of patients preferred self-collection (53%) to collection by health care professionals (21%), with 26% having no preference.^78^ In addition to enabling social distancing, a self-service model facilitates access to testing for those facing logistical challenges, such as lack of reliable childcare or ready access to transportation, while preserving the scarce capacities and resources of the health care system to treat severely ill patients.

## Vaccines and Treatments

Currently there is no fully FDA-approved vaccine for COVID-19, but accelerated work is underway to develop and produce hundreds of millions of vaccine doses in early 2021 (only 18 months).^79^ Historically, vaccine development requires an average of 10.7 years and yields market entry probability of only 6%.^80^ According to the World Health Organization, as of October 19, 2020, 154 vaccines were in development^81^ and 44 vaccines were in clinical trials,^79,82^ including 10 entering phase 3 trials.^82^ Updates on the development of vaccines can be found at the World Health Organization's *Draft Landscape of COVID-19 Vaccine Candidates* publication that is updated regularly.^82^ Additionally, the FDA provides an ongoing record of vaccines and COVID-19 treatments undergoing evaluation in the United States in their Coronavirus Treatment Acceleration Program database.^83^ As a COVID-19 vaccine becomes available, employers must consider accessibility, acceptability, and costs to the health plan to vaccinate the workforce. In addition, employers may consider strategies to encourage vaccination of the workforce in accordance with the EEOC.

### Clinical protocols and treatments

Because COVID-19 is a novel disease, clinical protocols and treatment guidelines are being developed and updated as credible information becomes available. For employers, an understanding of clinical protocols and treatments can help forecast costs for health care. A current analysis shows that a single symptomatic SARS-CoV-2 infection has a median direct medical cost of $3045 while a single hospitalized case has a median direct medical cost of $14,366 when only costs during the course of the infection are included.^84^

Current treatment for COVID-19 varies by the individual patient and severity of the symptoms. Currently, FDA-approved drugs for COVID-19 are lacking.^85,86^ In May, the investigational antiviral drug remdesivir was granted emergency use authorization by the FDA for the treatment of suspected or laboratory-confirmed COVID-19 in adults and children hospitalized with severe disease,^87^ and subsequently granted approval for the treatment of COVID-19 requiring hospitalization in October 2020.^88^ Yet, many drugs approved for other indications or investigational medications are being evaluated for the treatment of COVID-19 in clinical trials.^86,89^ Although no agent given before an exposure is known to be effective in preventing SARS-CoV-2 infection, clinical trials of hydroxychloroquine, chloroquine, and HIV protease inhibitors – despite potential – have yielded less promising results.^86,90,91^ In addition, medical providers may access and prescribe investigational drugs or agents approved or licensed for other indications through emergency use authorizations, Emergency Investigational New Drug applications, compassionate use, or expanded access programs with drug manufacturers, and/or off-label use.^86^ On August 23, 2020, the FDA issued an emergency use authorization for investigational convalescent plasma for the treatment of COVID-19 in hospitalized patients.^92^

### Workplace surveillance

Surveillance of integrated workplace-related COVID-19 information in a workplace surveillance system may facilitate contact tracing, isolation, and the timely decision-making necessary to protect employees and customers at the worksite. Successful surveillance systems facilitate prompt identification and isolation of infectious or potentially infectious individuals.^24^ Testing results enable contact tracing and isolation efforts to contain the spread of the virus and eventually may be used for treatments. (Further details of an employer testing and surveillance strategy is available in a companion paper by Plantes et al.^93^)

### Contact tracing

Should infections occur in the workplace, employers should develop protocols to trace and notify other employees who may have had contact with the infected individual. Protocols should maintain compliance with privacy and nondiscrimination laws. Thus, employer protocols should inform employees if they had close contact with someone who has or may have COVID-19, but may not reveal the identity of the individual.^94^

### Workplace metrics

Employers will need to develop mechanisms to monitor workplace infections to confirm the health and safety of the workplace. Protocols such as site closure may be required should observed increases in infections or symptoms (high incidence of elevated temperature) be apparent in a specific location. With sufficient testing and tracking, leaders can make more informed decisions about social distancing, on-site work, and transmission control measures.^95^ Key metrics for understanding the reach and severity of COVID-19 in a given area include number of new daily cases, tests per 100,000 people (testing rate), and percentage of tests that are positive (positivity rate).^95^

### Command center

Integration of internal and external data, such as community rates of infection, laboratory test data, symptoms, and privacy-protected employee data, to provide a population view/dashboard of trends in real time to a “command center” may inform local decision-making. Such a command center would facilitate monitoring of employee testing and evaluation of operational readiness levels and risks in specific locations or areas.

### Action

During the pandemic, employers may exclude individuals from the workplace if they have a medical condition, such as COVID-19, that would pose a direct threat to health or safety.^41^ This includes sending employees home if they have been diagnosed with COVID-19 or are displaying symptoms. If a person has or is suspected to have COVID-19, employers should close off any areas used for prolonged periods by the sick person and wait 24 hours before cleaning and disinfecting to reduce airborne exposure.^23^ Employers may instruct employees who were potentially exposed to stay home for 14 days, work remotely if possible, and self-monitor for symptoms.^23^ In the case of critical infrastructure employees, work may be permitted provided that the individuals remain asymptomatic and additional precautions (including continued screening, PPE, social distancing, and cleaning) are implemented to protect them and the community.^23^

## Anticipated Impact of COVID-19 on Health Benefits and Costs

### Shifts in health care coverage

As of August 2020, more than 50 million US employees (about 1 in 6 employees) had filed for unemployment since the coronavirus outbreak began in early March 2020.^96^ Unemployment not only poses economic hardships to employees and their families, but also affects access to health coverage that may have been provided previously by employers. Through May 2020, roughly 5.4 million Americans had lost health insurance coverage because of layoffs during the pandemic.^97^ Escalating unemployment rates are expected to cause a shift from commercial employer-sponsored health insurance to individual, Medicare, or Medicaid plans; millions may be left uninsured altogether.^98^ More than half of workers losing employer-sponsored coverage in Medicaid expansion states (37 states, including California, New England states, and New York) are expected to gain Medicaid coverage.^98^ In non-expansion states (13 states, including Florida, Texas, and Georgia), employees losing employer-sponsored coverage are more likely to become uninsured.^98^ As millions of lives migrate out of commercial plans, payers will experience a significant reduction in membership. Compensation in health coverage through increased Medicaid and Medicare coverage may promote accessibility to health care but strain state budgets, leading to restricted resources.^98^

During periods of economic contraction and unemployment, health care utilization decreases because of declining demand for medical care, as seen during the Great Recession (2007–2009).^99,100^ Even individuals with continued insurance may reduce discretionary spending on health care services and preventive measures^101^ because of fear of job loss, declining household income, and greater economic insecurity.

### Impact on health care costs

The pandemic has created a surge in demand on health care systems by requiring higher capacities for testing, PPE, hospital beds, trained staff, and ventilators. Although the need for COVID-19 testing and treatment may put upward pressure on health care spending, deferral and elimination of care are expected to have a larger influence on the annual cost of health care.^102^ Decreased health care utilization and the slowdown of elective surgeries put revenue pressure on hospital systems and health care providers. In 2020, COVID-19 could reduce employer health care costs by as much as 4% because of decreases in employees getting nonessential medical care.^103^ Although reductions vary by plan, they may be largest for commercial plans; Medicaid may experience increased costs from individuals shifting to the plan because of loss of employment.^102^ The American Hospital Association estimates that COVID-19 will cost America's hospitals and health systems an average of $50.7 billion per month from March to June 2020, mostly related to canceled surgeries, canceled outpatient treatment, and reduced emergency department services.^104^ Similarly, COVID-19 pandemic models estimate national reductions of between $140 billion and $375 billion in health care spend, based on deferred health care, through June 2020.^102^

Health plans and large employers remain uncertain about the expected impact of the pandemic on health care costs in 2021. An analysis by Covered California projected that premiums could increase in 2021 by anywhere from 4% to 40% because of the COVID-19 outbreak. The Health Research Institute offered 3 scenarios to guide employers and health plans to set 2021 health care premiums with health care cost trends ranging from 4% to 10%, depending on whether deferred health care is delivered in 2021.^105^ However, the impact of COVID-19 on health insurance premiums depends on when the spread of the virus is contained and if the pandemic extends into 2021. Insurance premiums for 2021 are based on expected costs during the 12-month period ending June 30, 2020, and are not supposed to include losses from prior years unless costs are not expected to persist. Thus, if the pandemic is limited to 2020, health insurance premiums may not increase as a result of COVID-19-related costs. Yet, projections of health care premiums also must consider the delayed and possibly compounded costs from deferred diagnoses and treatments, postponed elective surgeries, and interruptions in management of underlying health conditions.^105^

### Pharmaceutical supply chain

Although no major disruptions in pharmaceutical access have been observed, uncertainty remains in the future of the pandemic and its effect on the US drug supply.^106^ For employers, working with pharmacy benefits managers to monitor medication availability, quality, and pricing may help respond to changes. Both overseas manufacturing and the US distribution of medications are potential areas of concern.^106–109^ Surges in demand may result for medicines to treat respiratory disease and critical illness, or media-touted coverage for specific medications for emerging evidence of benefit, or other factors.^106^ Limited supplies of drugs used to treat both COVID-19, such as antimicrobials and sedatives, and other life-threatening conditions may trigger costs to rise,^110^ as has been seen with insulin.^111^ Drug shortages are expected to affect generic drugs, in particular, because of limited FDA-approved generic versions (only 1 or 2) available for sale in the United States.^110^ In addition, fewer new drugs for non-COVID-19-related treatment are expected to launch as a consequence of recent pharmaceutical development being focused on the novel coronavirus vaccinations.^112^ Health plan managers may work with health plans to develop an essential medicine strategy to ensure that priority health medications (including antibiotics, antivirals, antidiabetic agents, cardiovascular drugs, respiratory agents, contraceptives, mental health products, and analgesics) remain available in adequate amounts, with adequate quality and pricing.^106^ In addition, pharmacy benefits managers may take steps to limit stockpiling, increase access by extending current prior authorization criteria and easing refill limits, and establishing where COVID-19 treatments fall in the formularies.

## Adaptations in the Delivery of Care

### Telemedicine

In addition to health care costs, the COVID-19 pandemic also will likely affect how health care is delivered. Telemedicine provides opportunities to continue health care delivery while reducing exposure to infection in a hospital or clinic setting.^113^ The CARES Act facilitates accessibility to telemedicine by allowing high-deductible health plans to cover telemedicine services prior to a patient reaching the deductible, without regard to whether the services provided via telemedicine relate to COVID-19.^114^ Employers also can play a role in accessibility to health care through virtual options.^113^ For example, as noted earlier, health services and screening may be adapted to at-home collections. In addition, video consultations enable remote care for COVID-19 that includes automated triage, isolation of potentially contagious patients within care facilities, and electronic monitoring in intensive care units.^115^ Telemedicine for COVID-19 may be associated with high satisfaction among patients and staff, similar disease progression and use, and lower transaction costs compared with traditional clinic-based care.^116,117^

### Annual health screening

As employers are implementing physical-social distancing practices while employees continue essential work or return to on-site facilities, employers may question the feasibility of annual health screening events. Decisions to conduct annual screening must weigh the risks compared to benefits. For example, traditional on-site events, which attract crowds, may be substituted with specimen collections at patient-service centers that practice additional health and safety precautions, or through at-home self-collection kits (eg, by finger stick) for some screening tests. Interruptions to regular screening intervals may have undesired consequences. These include delayed identification of diseases for which early intervention can improve outcomes, and impaired management of chronic conditions such as type 2 diabetes and hypertension, which can lead to complications and higher health plan costs. Fall 2020 health screening also may foster the opportunity to add serology/antibody testing to test panels to gain insight on exposure to COVID-19 and the presence of SARS-CoV-2 antibodies.

### Annual influenza vaccine

As the COVID-19 pandemic is expected to overlap with seasonal influenza in the fall/winter of 2020–2021, the decision to offer and encourage or even mandate annual influenza vaccination is especially relevant. Not only may influenza vaccination reduce transmission of the disease in the workplace, but prevention of influenza also may reduce the strain on medical resources to preserve medical resource capacity to treat COVID-19. Mathematical models have shown that increasing influenza vaccination rates may facilitate management of respiratory outbreaks coinciding with the peak flu season.^118^ The health care industry has mandated influenza vaccination for health care workers to protect patients who may be at an elevated risk of complications from influenza.^119^ From a value perspective, influenza vaccination in adults is also cost-effective at $8000 to $39,000 per quality-adjusted life year gained (similar to other preventive services such as breast and colon cancer screening and hypertension management).^120^ Yet, employers who are considering mandatory influenza vaccinations must successfully navigate complex legal and ethical considerations and state laws. An alternative approach for employers may include strongly recommending employee influenza vaccinations without mandate, while also reducing presenteeism (coming to work sick).^121^

### Social determinants of health

During the COVID-19 pandemic, social determinants of health (SDOH) have emerged as key variables in susceptibility to infection and morbidity. SDOH include the conditions where people live, learn, work, and play that affect health risks and outcomes. The pandemic appears to be disproportionately affecting people from Black, Asian, and other minority ethnic communities, both in terms of hospitalizations and fatal outcomes.^122^ In regard to hospitalizations, in the United States, African Americans represent 33% of COVID-19 hospitalizations, despite making up only 18% of the total population studied.^122^ In New York City, for example, COVID-19-related death rates for Black or African American people (92.3 deaths per 100,000 population) and Hispanic or Latino people (74.3) are substantially higher than those of White (45.2) and Asian (34.5) people.^122^ Such disparities are likely attributable to both higher prevalence of chronic conditions and other societal factors.^122–125^ African Americans and Hispanic Americans have greater risk of underlying conditions such as obesity, diabetes, and hypertension,^126^ which have been associated with greater disease severity.

By understanding and addressing SDOH in their workforce and communities, employers may support the health of their working families.^127^ Housing instability inhibits families from being able to prioritize their health and the health of their children.^123^ In addition, when many people share a home, it is more difficult to social distance – a practice necessary to prevent transmission of the disease. Additionally, with virtual or closed schools, working parents also confront the expenses of childcare and meals that typically had been provided during the school day.^128^ As a result, food insecurity is expected to impact 16%-17% of lower-wage families.^128^ This may exacerbate future detrimental health consequences, as food, nutrition, social factors and health are linked multidirectionally.^129^ In addition, fear and unknowns associated with the virus have contributed to societal prejudice and discrimination toward some minority populations.^124,125^ Moreover, prejudice and discrimination have both unjustly led to disruptions in service and accessibility for individuals and sparked shame, stress, and stigma that impair infected individuals from reporting their illness and receiving appropriate and timely medical care.^125^

By realizing and playing a role in addressing SDOH, employers may support the long-term health of their working families. Employers may collect this information via focus groups, surveys,^127,130^ leveraging employee SDOH-relevant data from human resources (zip codes, income) and existing vendor partners (employee assistance program, financial programs, health plans), or available public health data.^130^ Evaluation of SDOH data may help employers to offer appropriate programs (financial security), referral services (food insecurity), and partner with the right health plans and programs to screen for and address SDOH in their delivery of care and services.

### Mental health: accommodating increased demand for behavioral health needs and access

Although social distancing and isolation are necessary to contain the virus and save lives, their impact on loneliness and mental health requires consideration. In fact, more than one third of Americans (36%) say that COVID-19 is having a serious impact on their mental health, according to a national poll by the American Psychiatric Association.^131^

Employers can play a role both directly, through employee interactions, and indirectly, by offering programs and support to accommodate increased mental health care needs. Managers can support employee interactions directly by ensuring that each employee receives daily outreach during the work week, through a supervisor or buddy system, just to maintain social contact.^132^ In addition, people managers in the workplace play an important role in transparency and communication to help foster higher perceived knowledge–an important factor associated with emotional well-being during the pandemic.^133^ Positive psychology in the workplace may be fostered through both clear communications of the decisions related to the business continuity plan of the organization during the pandemic and by involving employees in the preparation of the post-pandemic business plan.^134^ Such practices may reduce employees' level of stress, foster positive attitudes, and reinforce team cohesion.^134^

### Managing interrupted and delayed care for chronic conditions

Ongoing health management can prevent complications or exacerbations of chronic disease and reduce preventable hospitalization. Yet, individuals with chronic conditions may experience disruptions in chronic care management because of inability to access health care facilities for routine care and medicines management during the COVID-19 pandemic.^135^ In fact, 69% of patients with chronic conditions surveyed reported some impact on their ability to manage their current conditions.^136^ Disruptions in routine care may present particularly detrimental consequences for diabetes management, where reduced HbA1c testing frequency or poor medication adherence may result in poorer blood glucose control,^137^ more hospitalizations,^138^ and associated complications^139^ and medical costs.^140^ HbA1c testing volumes at a large clinical reference laboratory declined by 66% in the first 8 weeks of March and April 2020, indicating disruptions in diabetes monitoring with subsequent blood glucose control consequences.^141^ The economic impact of reduced hours, furloughs, and unemployment also may present a financial barrier to medication adherence for glycemic control, further compounding poor condition management.^135^ In addition, the acute psychological stress from the pandemic may further contribute to increased glucose concentrations in patients with type 2 diabetes.^142^ The response to the COVID-19 pandemic also may result in delayed diagnosis of medical conditions. For example, the numbers of patients with newly identified cancer decreased during the early weeks of the pandemic, by 25% to 52% depending on the type of cancer.^143^

Disruption in care, monitoring, and therapy adjustment for individuals with chronic conditions can result in significant consequences, complications, hospitalizations, and costs. Such consequences highlight the need for innovative solutions to make chronic care management more convenient and accessible for employees during the pandemic. Access to health screenings and condition monitoring may be preserved via alternative methods such as self-collection models coupled with virtual care.^144,145^ Videoconferencing technology enables “in-person” visits for the management of chronic diseases such as cardiovascular diseases, diabetes,^116^ and psychotherapy,^117^ while avoiding the associated fear of COVID-19 exposure. Videoconferencing technology has demonstrated feasibility and benefit^116,117^ with user satisfaction^117^ and clinical outcomes similar to those of traditional face-to-face therapy.^117^

## Conclusions

COVID-19 has dramatically changed the outlook of employee population health management for employers in 2020. As business continues in a new normal, employees return to work, and workforces adjust to changing business demands, health programs must take on a new challenge in maintaining the health of the workforce. In recent years, before the COVID-19 pandemic, chronic diseases were considered the main modifiable drivers of morbidity and mortality and were the main focus of health promotion efforts. Now, employers must maintain health management and cost mitigation strategies focused on chronic disease risk factors while also containing the spread of a novel infectious disease. Until vaccination becomes available, return to work practices focused on mitigating the spread of COVID-19 through safety practices, testing, and surveillance enable employers to monitor and make informed decisions regarding the near-term health of the workforce population. At the same time, employers can anticipate the impact of COVID-19 on health benefits and costs (including adaptations in delivery of care, social and behavioral health needs, and managing interrupted care for chronic conditions), while recognizing that chronic conditions and behavioral health needs cannot be delayed. In leveraging innovative technologies such as telemedicine and remote testing options while appreciating the rising needs for mental health services and social factors related to both chronic and infectious disease, employers may be better equipped to navigate the new health care environment.
